# Chemosensitizer Effects of Cisplatin- and 5-Fluorouracil-Treated Hepatocellular Carcinomas by Lidocaine

**DOI:** 10.3390/ijms26157137

**Published:** 2025-07-24

**Authors:** Teng-Wei Chen, Hsiu-Lung Fan, Shu-Ting Liu, Shih-Ming Huang

**Affiliations:** 1 Division of General Surgery, Department of Surgery, Tri-Service General Hospital, National Defense Medical Center, Taipei City 114, Taiwan; tengweichen@yahoo.com.tw (T.-W.C.); doc20450@ndmctsgh.edu.tw (H.-L.F.); 2 Department of Biochemistry, National Defense Medical Center, Taipei City 114, Taiwan; shuting0719@gmail.com

**Keywords:** chemotherapy, lidocaine, hepatic artery infusion chemotherapy, reactive oxygen species, endoplasmic reticulum stress

## Abstract

Approximately 90% of liver cancer cases are classified as hepatocellular carcinomas (HCCs), with chemotherapy and immunotherapy being the most recommended treatment options. While conventional chemotherapy specifically targets rapidly dividing cancer cells, it can also impact on healthy cells that are proliferating quickly. This collateral damage to healthy cells, along with the potential for cancer cells to develop resistance, presents significant challenges for conventional chemotherapy in liver cancer patients. Hepatic artery infusion of chemotherapy (HAIC) generally leads to reduced toxicity and fewer side effects. The process of catheter insertion is usually performed under local anesthesia, with lidocaine being the preferred choice to combine with various chemotherapeutics in HCC treatment. In our study, we explored the effects of repurposing lidocaine in combination with cisplatin or 5-fluorouracil (5-FU) on two HCC cell lines, HepG2 and Hep3B. Our cytotoxicity analysis revealed that lidocaine functions as a chemosensitizer for cisplatin and 5-FU in both HepG2 and Hep3B cells. Specifically, we observed an increase in the subG1 population and a reduction in cytosolic reactive oxygen species in cisplatin- or 5-FU-treated HepG2 and Hep3B cells. Interestingly, lidocaine selectively decreased the reduced/oxidized glutathione ratio in cisplatin- or 5-FU-treated HepG2 cells but not in Hep3B cells. Furthermore, lidocaine induced endoplasmic reticulum stress, apoptosis, mitochondrial membrane depolarization, lipid peroxidation, and autophagy while suppressing cellular proliferation HepG2 and Hep3B cells. In conclusion, our study demonstrates the synergistic potential of combining lidocaine with cisplatin or 5-FU for the treatment of HCC, indicating that lidocaine may serve as an effective chemosensitizer. These findings highlight a new clinical advantage of using repurposing lidocaine as a chemosensitizer in the current HAIC procedure, suggesting that this combination warrants further exploration through rigorous clinical trials. In the future, we can better optimize therapeutic regimens, potentially leading to improved patient outcomes in HCCs.

## 1. Introduction

Hepatocellular carcinomas (HCCs) account for 90% of primary liver cancers and can be molecularly classified into two subtypes, namely proliferation and non-proliferation, based on DNA somatic alterations, epigenetic features, biological phenotypes, and clinical characteristics [1,2,3]. Most patients with HCC are diagnosed at advanced stages of the disease, and conventional systemic chemotherapy offers limited survival benefits. Therefore, molecularly targeted therapies, including various kinase inhibitors, along with immunotherapies such as immune-checkpoint inhibitors, are currently the most recommended treatment options. The growing number of clinical trials exploring combinations of targeted therapies and immunotherapies aims to enhance clinical efficacy and reduce drug resistance in HCC patients [4].

Given the limited survival benefits associated with conventional chemotherapy, agents like cisplatin, 5-fluorouracil (5-FU), and doxorubicin have demonstrated some effectiveness in treating liver cancer, though they often only manage to shrink a small number of tumors. In certain cases, chemotherapy may be recommended prior to surgery to reduce tumor size and decrease the risk of recurrence. While these drugs specifically target rapidly dividing cancer cells, they also impact healthy cells that divide quickly, such as those in the intestinal lining, mouth, bone marrow, and hair follicles. The adverse effects on healthy cells and the potential for cancer cells to develop resistance pose significant challenges for liver cancer patients undergoing chemotherapy [5,6,7]. Combining drugs and modifying administration methods offer new opportunities for improving treatment outcomes [8,9]. Hepatic artery infusion of chemotherapy (HAIC) enables a higher concentration of chemotherapeutic agents to reach the tumor, often proving more effective than conventional systemic chemotherapy [10,11,12]. HAIC typically results in reduced toxicity and fewer side effects. Various chemotherapeutic agents have been utilized individually or in combinations; however, the effectiveness of this treatment approach is still a matter of ongoing debate.

Repurposing Food and Drug Administration (FDA)-approved drugs could significantly shorten development time and reduce costs compared to de novo drug discovery [13]. Recently, drug repurposing has emerged as a powerful and efficient strategy for identifying and developing novel anticancer agents [14]. For instance, several commonly prescribed cardiovascular medications, including cardiac glycosides, statins, and β-blockers, have exhibited additional therapeutic effects that could benefit cancer treatment and prevention [15,16,17]. Catheter insertion for HAIC typically occurs under local anesthesia, utilizing lidocaine at the arterial puncture and port-a-cath subcutaneous pocket sites [18]. Furthermore, the surgical removal of the primary tumor is a critical component of treatment for various cancer types. Therefore, the careful selection of anesthetic and analgesic agents during surgery is essential to minimize the risk of cancer recurrence. This better choice not only enhances perioperative safety but also improves postoperative outcomes [19,20]. Recent studies indicate that local anesthetics may be repurposed as chemosensitizers or synergistic therapies in oncology [21,22,23,24]. It is imperative to explore collaborative strategies that integrate chemotherapeutics with local anesthetics to enhance treatment efficacy and mitigate resistance in HCCs. Consequently, investigating the repurposing potential of lidocaine in combination with cisplatin or 5-FU presents an intriguing area for further study.

The primary goal of combination therapy is to achieve synergistic effects, allowing for dosage reduction and a subsequent decrease in toxicity. Synergism quantification methods are typically classified into effect-based and dose-effect based approaches. However, existing methods face several limitations, including the ambiguous use of the term “synergy”, the lack of a standard reference model for evaluating synergism, and various practical and ethical constraints in dose optimization [25,26,27]. In this study, we employed the Chou–Talalay method, which is the most commonly used approach for quantifying drug combinations, particularly for identifying synergistic interactions [28,29]. Chou–Talalay’s combination index (CI) theory is derived from the median-effect equation, based on the unified theory of mass-action law. The CI can then be computed using CalcuSyn software version 2 to generate isobolograms (ED_50_). Isobologram analysis complements algebraic assessments with an intuitive, flexible, and widely accepted graphical approach [30]. Generally, a CI value of less than 1 indicates a synergistic effect, whereas a value greater than 1 suggests an additive effect.

In addition to DNA somatic alterations and epigenetic features, viral infections such as hepatitis B virus (HBV) are also contributors to HCC development. HepG2 and Hep3B cell lines are frequently utilized for in vitro toxicity studies related to HCCs [31,32]. HepG2 cells possess wild-type p53 and are HBV-negative and non-tumorigenic, while Hep3B cells are p53-deficient, HBV-positive, and tumorigenic. In this study, we investigated the cytotoxic effects of lidocaine on both HepG2 and Hep3B cell lines. We subsequently assessed whether lidocaine could work synergistically with cisplatin or 5-FU. Finally, we evaluated the potential functional impacts of lidocaine on cell proliferation, cell cycle profiles, oxidative responses, autophagy, and mitochondrial dysfunction. Our study aimed to clarify the synergistic interactions between lidocaine and either cisplatin or 5-FU, as well as any differences in responsiveness between HepG2 and Hep3B cells. Ultimately, our findings may contribute to the development of a novel combination therapy approach for HCCs, leveraging lidocaine’s dual functional roles in both surgical and drug treatments for liver cancer.

## 2. Results

### 2.1. The Synergistic Effect of Lidocaine Combined with Cisplatin or 5-FU in Human Hepatocellular Carcinoma Cell Lines

**Figure 1:**
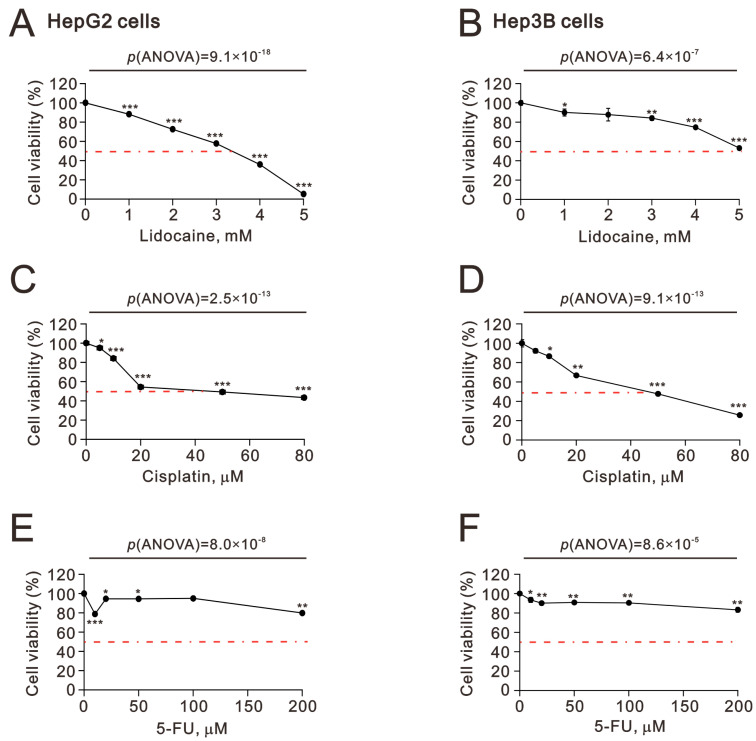
Effects of lidocaine, cisplatin, and 5-FU on cell viability in HepG2 and Hep3B cells. (**A**,**C**,**E**) HepG2 and (**B**,**D**,**F**) Hep3B cells were treated with the specified concentrations of lidocaine (**A**,**B**), cisplatin (**C**,**D**), and 5-FU (**E**,**F**) for 24 h. Cell metabolic activity was assessed using the MTT assay. The results are representative of three independent experiments. The dashed red line is the level of 50% cell viability. Bars depict the means ± SDs of three independent experiments. * *p* < 0.05; ** *p* < 0.01; *** *p* < 0.001 (Student’s *t*-tests). Original data are presented in the Supplementary Material (Figures S1 and S2).

We began by assessing the cytotoxic effects of lidocaine, cisplatin, and 5-FU on HCC cell lines, specifically HepG2 and Hep3B (Figure 1). Except for 5-FU treatment, the IC_50_ values for lidocaine and cisplatin were found to be 3.5 mM and 45 μM in HepG2 cells (*p*(ANOVA) = 9.1 × 10^−18^ and *p*(ANOVA) = 2.5 × 10^−13^) (Figure 1A,C) and 5 mM and 50 μM in Hep3B cells (*p*(ANOVA) = 6.4 × 10^−7^ and *p*(ANOVA) = 9.1 × 10^−13^) (Figure 1B,D). Our findings consistently indicate that compared to cisplatin, 5-FU is not an effective chemotherapy option for HCCs (Figure 1E,F). Lidocaine exhibited cytotoxic effects in HCCs.

**Figure 2:**
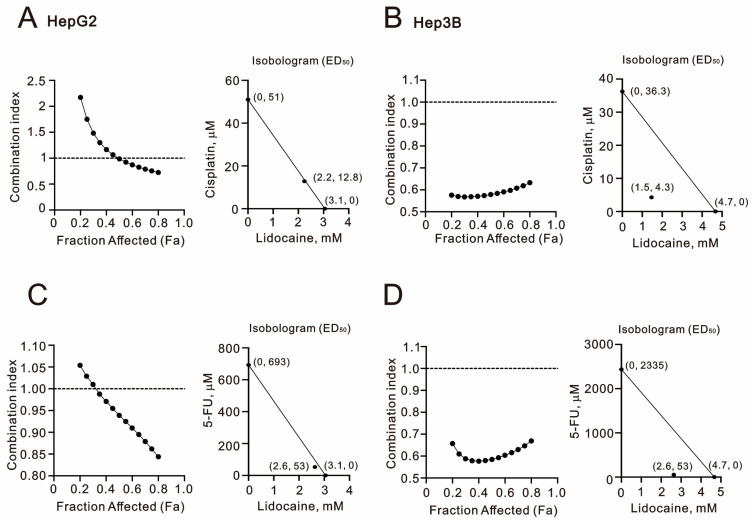
Combination index of lidocaine with cisplatin and 5-FU in HepG2 and Hep3B cells. (**A**,**C**) HepG2 and (**B**,**D**) Hep3B cells were treated with varying concentrations of lidocaine (0, 1, 2, 3, 4, 5, 6, or 7 mM) in combination with cisplatin (**A**,**B**) at doses of 0, 0.156, 0.3125, 0.625, 1.25, 2.5, 5, 10, 20, and 40 µM or with 5-FU (**C**,**D**) at doses of 0, 5, 10, 20, 50, 100, 200, 250, 350, and 500 µM. Cell viability was assessed using the MTT assay. The combination index for lidocaine in conjunction with either cisplatin (**A**,**B**) or 5-FU (**C**,**D**) was calculated. Additionally, isobolograms (ED_50_) for the combinations of lidocaine with cisplatin or 5-FU were determined using CalcuSyn software. Original data and analytical results are provided in the Supplementary Material (Figure S3).

Combination therapy is becoming increasingly popular in the treatment of HCCs. We conducted combination index analyses by treating HepG2 and Hep3B cells with lidocaine alongside cisplatin or 5-FU. An index value below 1 indicates that combination therapy is synergistic. Our results demonstrated that the combination of lidocaine with either cisplatin or 5-FU in both HepG2 and Hep3B cells exhibited synergistic effects. Notably, the synergistic effects were most pronounced when lidocaine was combined with 5-FU in both cell lines (Figure 2). In HepG2 cells, the lidocaine concentration was reduced from 3.1 mM to 2.2 mM, while the IC_50_ of cisplatin decreased from 51 μM to 12.8 μM, and that of 5-FU decreased from 693 μM to 52.7 μM (the lidocaine concentration was 2.6 mM) (Figure 2A,C). In Hep3B cells, the lidocaine concentration was lowered from 4.7 mM to 1.5 mM, and the IC_50_ values for cisplatin and 5-FU dropped from 36.3 μM to 4.3 μM and from 2335 μM to 53 μM, respectively (the lidocaine concentration was 2.6 mM for 5-FU) (Figure 2B,D). Our findings suggest that the combination of cisplatin or 5-FU with lidocaine could reduce the risk of side effects and drug resistance in HCC patients treated with cisplatin or 5-FU.

### 2.2. The Potential Mechanisms of Synergistic Effect of Lidocaine Combined with Cisplatin or 5-FU in Human Hepatocellular Carcinoma Cell Lines

**Figure 3:**
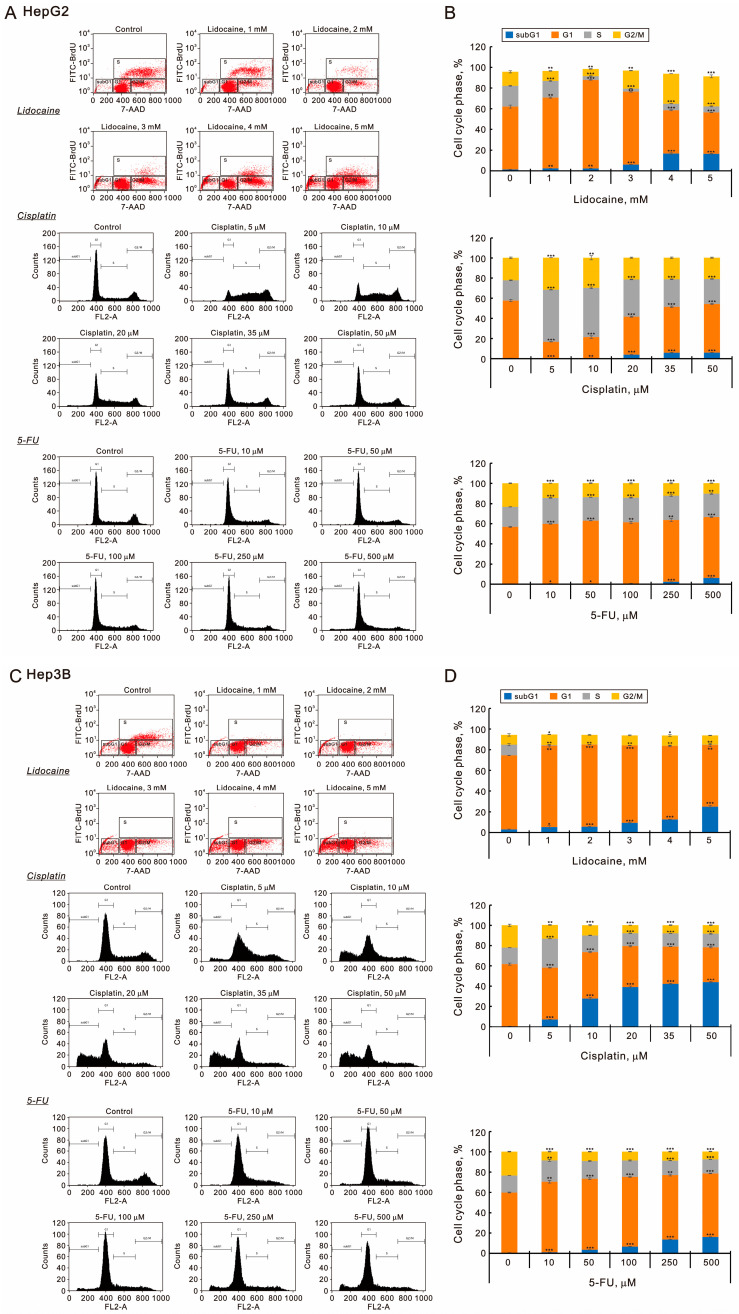
Effects of lidocaine, cisplatin, and 5-FU on cell cycle profile in HepG2 and Hep3B cells. Panels (**A**,**B**) and (**C**,**D**) show HepG2 and Hep3B cells treated with specified concentrations of lidocaine, cisplatin, and 5-FU for 24 h. (**A**,**C**) The cell cycle profiles were analyzed using flow cytometry with 7-amino-actinomycin D (for lidocaine) and PI (for cisplatin and 5-FU) staining for DNA labeling. The resulting cell cycle profiles were determined, with outcomes representing three independent experiments. (**B**,**D**) Bars depict the means ± SDs of three independent experiments. * *p* < 0.05; ** *p* < 0.01; *** *p* < 0.001 (Student’s *t*-tests).

To further investigate the cytotoxic effects of lidocaine, cisplatin, and 5-FU, we analyzed the cell cycle distributions in HepG2 and Hep3B cells (Figure 3). All three agents—lidocaine, cisplatin, and 5-FU—were found to increase the populations in the subG1 phase in both cell lines. Lidocaine significantly reduced the proportion of cells in the S phase while initially increasing and then decreasing the populations of the G1 phase in both HepG2 and Hep3B cells (Figure 3A–D). In HepG2 cells, lidocaine first decreased and then increased the G2/M phase populations, whereas it had no notable effect on Hep3B cells (Figure 3B,D). Lower concentrations of cisplatin decreased the G1 phase populations and increased those in the S phase in both cell lines. Conversely, higher concentrations of cisplatin inhibited the decrease in G1 and the increase in S phase populations in HepG2 cells, while they enhanced the decrease in G1 and G2/M phase populations in Hep3B cells. Additionally, 5-FU increased the G1 phase population while decreasing the G2/M phase population in both cell lines. In HepG2 cells, 5-FU raised the S phase population, whereas in Hep3B cells, lower concentrations increased the S phase population, and higher concentrations decreased it.

**Figure 4:**
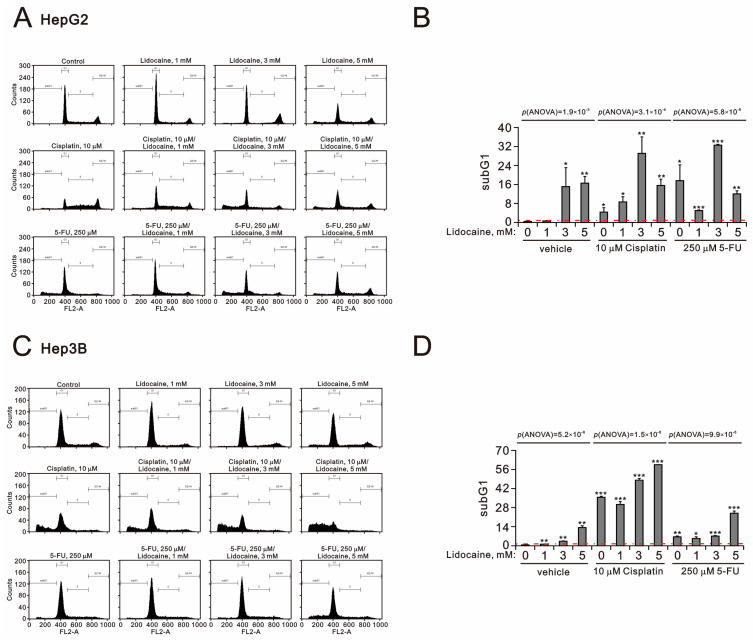
Effects of lidocaine, cisplatin, and 5-FU on cell apoptotic in HepG2 and Hep3B cells. (**A**,**B**) HepG2 and (**C**,**D**) Hep3B cells were treated with the specified concentrations of lidocaine in combination with cisplatin (10 µM) and 5-FU (250 µM) for 24 h. (**A**,**C**) The cell cycle profiles were then analyzed using flow cytometry with PI staining, as shown in panels. (**B**,**D**) The dashed red line is the level for the vehicle control. Bars depict the means ± SDs of three independent experiments. * *p* <0.05; ** *p* < 0.01; *** *p* < 0.001 (Student’s *t*-tests).

We also investigated the effects of combining lidocaine with cisplatin or 5-FU on the subG1 populations in both HepG2 and Hep3B cells (Figure 4A–D). Our findings revealed that there was an increase in subG1 populations at concentrations of 3 mM lidocaine, 10 μM cisplatin, or 250 μM 5-FU in both cell lines. Synergistic effects of lidocaine with either cisplatin or 5-FU were observed, with 3 mM lidocaine combined with 10 μM cisplatin or 250 μM 5-FU in HepG2 cells (*p*(ANOVA) = 1.9 × 10^−3^, *p*(ANOVA) = 3.1 × 10^−4^, and *p*(ANOVA) = 5.8 × 10^−5^) as well as 5 mM lidocaine combined with 10 μM cisplatin or 250 μM 5-FU in Hep3B cells (*p*(ANOVA) = 5.2 × 10^−8^, *p*(ANOVA) = 1.5 × 10^−8^, and *p*(ANOVA) = 9.9 × 10^−8^) (Figure 4B,D).

**Figure 5:**
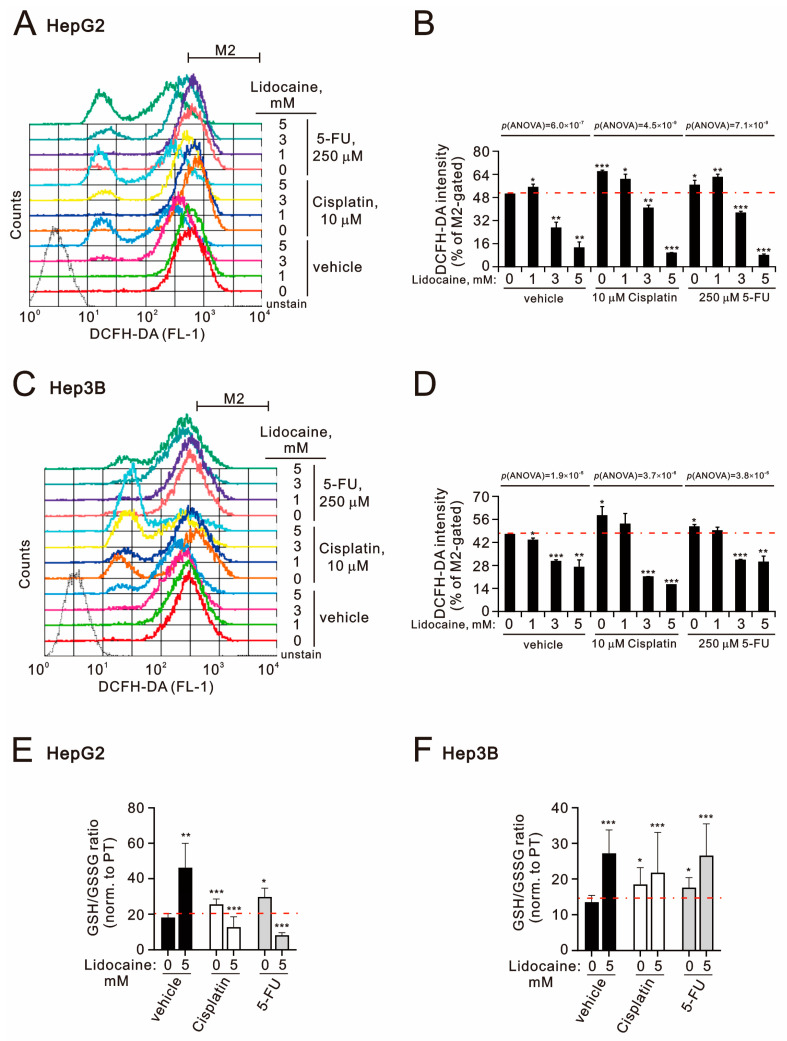
Effects of lidocaine, cisplatin, and 5-FU on cytosolic ROS and GSH/GSSG status in HepG2 and Hep3B cells. (**A**,**B**,**E**) HepG2 and (**C**,**D**,**F**) Hep3B cells were treated with the specified concentrations of lidocaine in combination with cisplatin (10 µM) and 5-FU (250 µM) for 24 h. Panels ((**A**,**B**) for HepG2 cells) and ((**C**,**D**) for Hep3B cells) display the DCFH-DA intensity measurements for cytosolic ROS levels. (**E**,**F**) The intracellular GSH/GSSG levels were determined using the GSH/GSSG-Glo™ assay and are presented in panels. The results are representative of three independent experiments. The dashed red line is the level for the vehicle control. Bars depict the means ± SDs of three independent experiments. * *p* < 0.05; ** *p* < 0.01; *** *p* < 0.001 (Student’s *t*-tests).

To elucidate the synergistic effect of lidocaine combined with cisplatin or 5-FU, we assessed the levels of reactive oxygen species (ROS) using DCFH-DA intensity and evaluated the antioxidant capacity by measuring the reduced/oxidized glutathione (GSH/GSSG) ratio in HepG2 and Hep3B cells (Figure 5). The DCFH-DA intensities increased with cisplatin and 5-FU treatment in both cell lines; however, lidocaine concentrations above 3 mM resulted in decreased intensity (Figure 5A–D). Furthermore, 5 mM lidocaine diminished ROS levels induced by cisplatin or 5-FU, exhibiting lower intensity than when lidocaine was used alone in HepG2 cells (*p*(ANOVA) = 6.0 × 10^−7^, *p*(ANOVA) = 4.5 × 10^−9^, and *p*(ANOVA) = 7.1 × 10^−9^) (Figure 5B). In Hep3B cells, lidocaine was similarly found to suppress ROS levels in the presence of cisplatin, with intensity lower than that of lidocaine alone (*p*(ANOVA) = 1.9 × 10^−5^, *p*(ANOVA) = 3.7 × 10^−6^, and *p*(ANOVA) = 3.8 × 10^−6^) (Figure 5D). Notably, lidocaine reduced 5-FU-induced ROS levels below baseline. We quantified the antioxidant effects of lidocaine, cisplatin, and 5-FU by measuring the GSH/GSSG ratio in HepG2 and Hep3B cells. All three compounds elevated the GSH/GSSG ratio in both cell lines (Figure 5E,F). However, lidocaine negated the elevation of this ratio caused by cisplatin or 5-FU in HepG2 cells but had no significant effect on Hep3B cells. Superoxide anion, hydroxyl radical, and hydrogen peroxide are three primary ROS produced during cellular metabolism [33]. The application of DCFH-DA serves to detect hydrogen peroxide in the cytoplasm. The discrepancies observed in ROS levels and GSH/GSSG ratios in Hep3B cells (Figure 5D,F) may arise from the limited accessibility to free radicals, which are distributed across various subcellular compartments when using DCFH-DA. Additionally, the GSH/GSSG ratio does not fully encompass the entire range of antioxidant capacities. To address this controversy, a more detailed investigation is necessary.

### 2.3. The Effects of Lidocaine, Cisplatin, and 5-FU on Autophagy, Cell Cycle Progression, Endoplasmic Reticulum Stress, and Signaling Proteins in Human Hepatocellular Carcinoma Cell Lines

**Figure 6:**
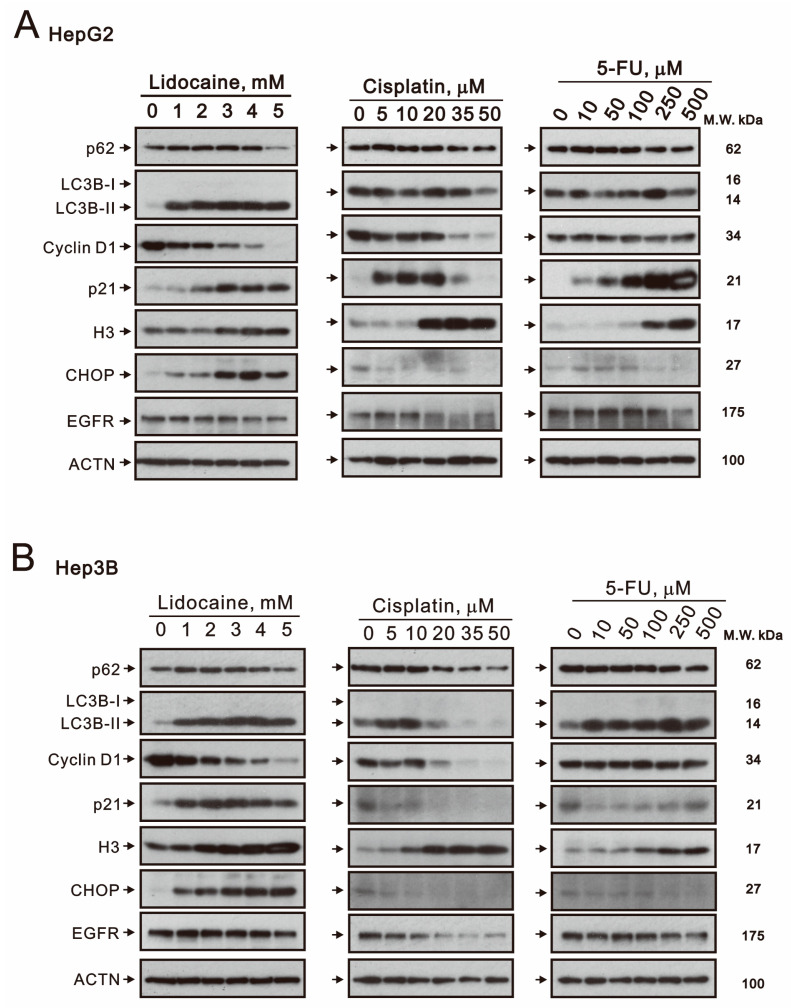
Effects of lidocaine, cisplatin, and 5-FU on autophagy, cell cycle profile, ER stress, and signaling related proteins in HepG2 and Hep3B cells. Panels (**A**,**B**) show HepG2 and Hep3B cells treated with specified concentrations of lidocaine, cisplatin, and 5-FU for 24 h. The cell lysates, containing 30 µg of total protein, were analyzed by Western blotting using antibodies targeting the indicated proteins. ACTN served as the loading control for protein levels.

Autophagy was induced by lidocaine in breast, endometrial, and bladder cancer cells [20,34,35]. The mechanism of action of conventional chemotherapeutics, including cisplatin and 5-FU, interferes with DNA replication by targeting various stress responses and signaling pathways [5,6,7]. Western blot analysis revealed that lidocaine increased the levels of autophagy-related marker microtubule-associated protein light chain 3B-II (LC3BII), cell cycle progression marker p21, histone H3, and endoplasmic reticulum (ER) stress marker CCAAT-enhancer-binding protein homologous protein (CHOP) while decreasing the levels of autophagic marker p62, cell cycle progression marker cyclin D1, and signaling protein EGFR in a dose-dependent manner in both HepG2 and Hep3B cells (Figure 6A,B). In HepG2 cells, cisplatin increased the levels of p21 and histone H3 while decreasing p62, LC3BII, cyclin D1, CHOP, and epidermal growth factor receptor (EGFR). Aside from a notable increase in p21, 5-FU exhibited similar effects as cisplatin (Figure 6A). In Hep3B cells, cisplatin elevated histone H3 levels while reducing p62, LC3BII, cyclin D1, p21, CHOP, and EGFR levels (Figure 6B). 5-FU increased the levels of autophagic marker LC3BII and histone H3 but decreased those of p62, cyclin D1, p21, CHOP, and EGFR.

### 2.4. The Cytotoxic Effects of Lidocaine in Human Hepatocellular Carcinoma Cell Lines

**Figure 7:**
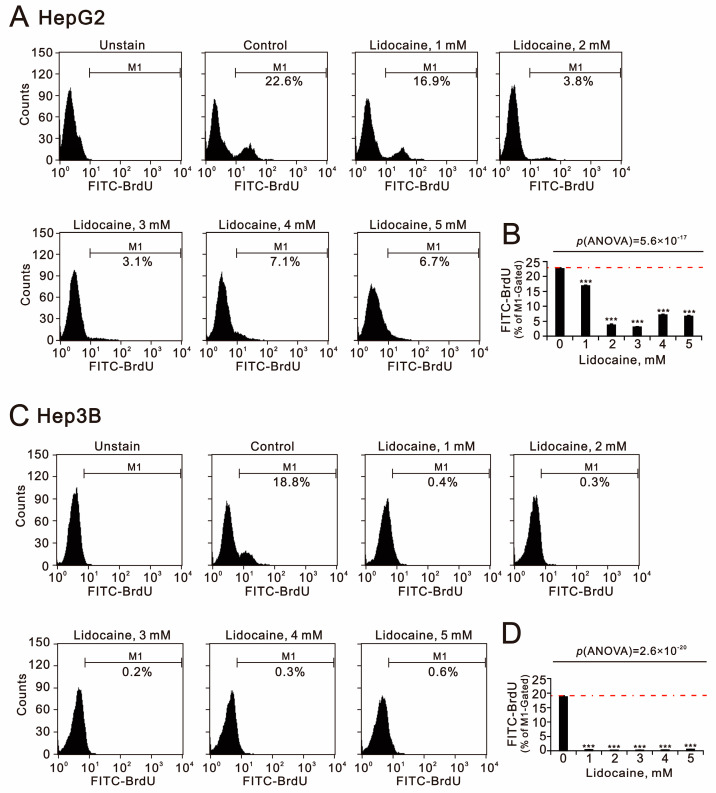
Effects of lidocaine on cell proliferation in HepG2 and Hep3B cells. (**A**,**B**) HepG2 and (**C**,**D**) Hep3B cells were treated with the specified concentrations of lidocaine for 24 h. (**A**,**C**) Cell proliferation was assessed using flow cytometry with FITC-BrdU incorporation, as shown in panels. (**B**,**D**) The dashed red line is the level for the vehicle control. Bars depict the means ± SDs of three independent experiments. *** *p* < 0.001 (Student’s *t*-tests).

The repurposing of lidocaine primarily highlights its various functional roles in anti-cancer properties, including the suppression of cellular proliferation, invasion, and migration as well as the induction of apoptosis [21,22,23,36]. We first utilized the bromodeoxyuridine (BrdU) assay to assess cellular proliferation (Figure 7A,C), and our data indicated that lidocaine effectively suppressed proliferation in both HepG2 and Hep3B cells (*p*(ANOVA) = 5.2 × 10^−17^ and *p*(ANOVA) = 2.6 × 10^−20^) (Figure 7B,D). Notably, the suppression was more pronounced in Hep3B cells.

**Figure 8:**
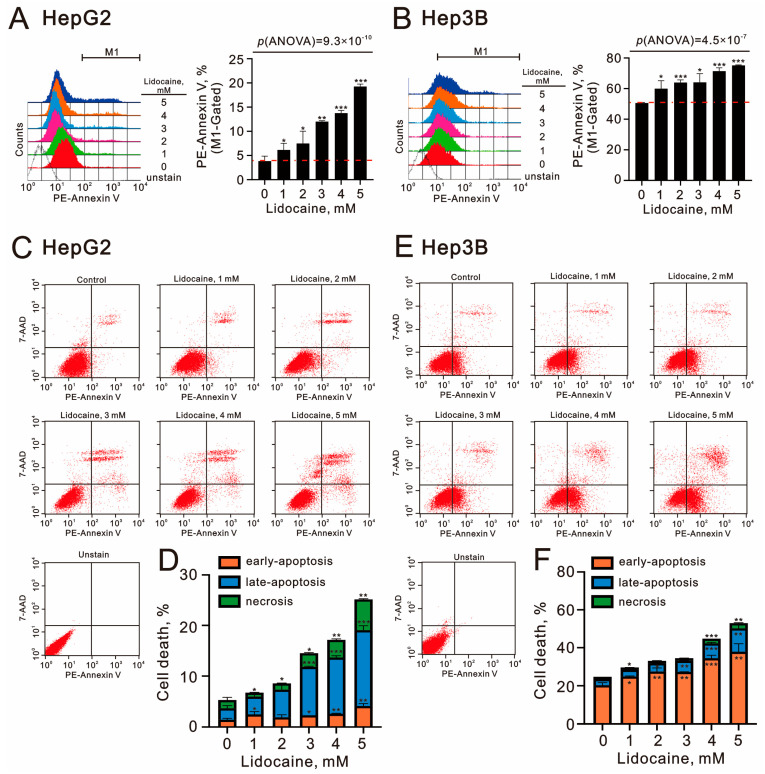
Effects of lidocaine on cell apoptosis in HepG2 and Hep3B cells. (**A**,**C**,**D**) HepG2 and (**B**,**E**,**F**) Hep3B cells were treated with the specified concentrations of lidocaine for 24 h. Panels (**A**,**B**) illustrate cellular apoptosis, measured using PE-Annexin V staining along with 7-AAD. The PE-Annexin V fluorescence intensity from the vehicle control was used as the baseline for M1 gating. In panels (**C**,**E**), early apoptotic cells are characterized as PE-Annexin V-positive and 7-AAD-negative, while late apoptotic cells are PE-Annexin V-positive and 7-AAD-positive. Necrotic cells are identified as PE-Annexin V-negative and 7-AAD-positive. The dashed red line is the level for the vehicle control. (**D**,**F**) Bars depict the means ± SDs of three independent experiments. * *p* < 0.05; ** *p* < 0.01; *** *p* < 0.001 (Student’s *t*-tests).

Next, we employed Annexin V and 7-amino-actinomycin D (7-AAD) to evaluate early and late apoptotic events as well as necrosis in HepG2 and Hep3B cells treated with lidocaine (Figure 8). We observed that the percentage of PE-Annexin V-positive cells increased with higher doses of lidocaine in both cell lines (*p*(ANOVA) = 9.3 × 10^−10^ and *p*(ANOVA) = 4.5 × 10^−7^) (Figure 8A,B). However, the baseline levels of PE-Annexin V were significantly higher in Hep3B cells compared to HepG2 cells. Additionally, the effects of lidocaine were characterized by an increase in late apoptotic and necrotic cells in HepG2 cells, while in Hep3B cells, both early and late apoptotic cells were noted (Figure 8C,D for HepG2 cells; Figure 8E,F for Hep3B cells). The observed cytotoxicity may be mediated by the suppression of cellular proliferation and the induction of apoptosis in HCC cell lines.

**Figure 9:**
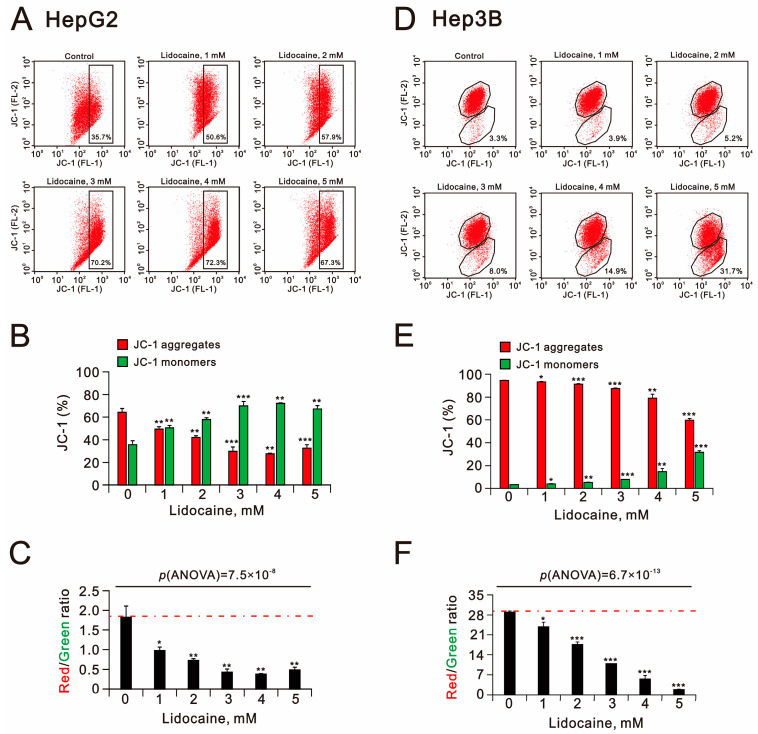
Effects of lidocaine on mitochondrial membrane potential in HepG2 and Hep3B cells. (**A**–**C**) HepG2 and (**D**–**F**) Hep3B cells were treated with specified concentrations of lidocaine for 24 h. (**A**,**D**) Mitochondrial membrane potential was assessed using flow cytometry with JC-1 staining, which detected the monomer (green fluorescence) in the cytosol and aggregate (red fluorescence) forms of the dye in the mitochondria. In panels (**B**,**E**), the percentages of red and green fluorescence intensities are depicted. Panels (**C**,**F**) show the measured and plotted red/green fluorescence intensity ratios. The dashed red line is the level for the vehicle control. Bars depict the means ± SDs of three independent experiments. * *p* < 0.05; ** *p* < 0.01; *** *p* < 0.001 (Student’s *t*-tests).

Mitochondrial membrane depolarization, lipid peroxidation, and autophagy are key cytotoxic mechanisms associated with HCC therapies [37,38]. To investigate the effect of lidocaine on mitochondrial membrane potential, we utilized the JC-1 dye in HepG2 and Hep3B cells (Figure 9A–D). Increasing concentrations of lidocaine correspondingly elevated the percentage of green fluorescence from the JC-1 dye, indicating polarization loss in the mitochondrial membranes of both cell types (Figure 9B,E). The red-to-green ratio significantly decreased, with a more notable reduction observed in Hep3B cells compared to HepG2 cells (*p*(ANOVA) = 7.5 × 10^−8^ and *p*(ANOVA) = 6.7 × 10^−13^) (Figure 9C,F).

**Figure 10:**
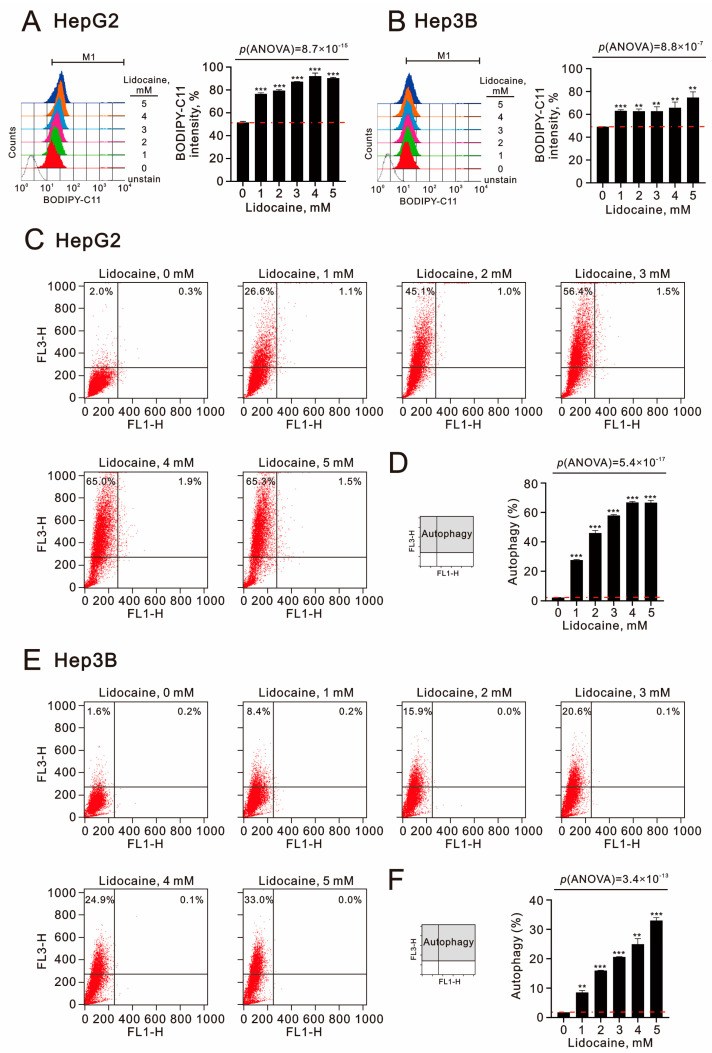
Effects of lidocaine on lipid peroxidation and autophagy in HepG2 and Hep3B cells. (**A**,**C**,**D**) HepG2 and (**B**,**E**,**F**) Hep3B cells were treated with specified concentrations of lidocaine for 24 h. (**A**,**B**) Lipid peroxidation was evaluated using flow cytometry with BODIPY-C11 staining, and the corresponding fluorescence intensity is presented in panels. (**C**,**E**) For autophagic cell measurement, acridine orange (1 µg/)mL staining was performed using flow cytometry. (**D**,**F**) The intensity of the red fluorescence (*y*-axis, FL3-H) was directly proportional to the acidity level and the volume of acidic vesicular organelles, including autophagic vacuoles. The dashed red line is the level for the vehicle control. Bars depict the means ± SDs of three independent experiments. ** *p* < 0.01; *** *p* < 0.001 (Student’s *t*-tests).

To assess lipid peroxidation induced by lidocaine, we utilized BODIPY-C11 dye in HepG2 and Hep3B cells. Lidocaine treatment resulted in increased lipid peroxidation, with a higher fold induction observed in HepG2 cells compared to Hep3B cells (*p*(ANOVA) = 8.7 × 10^−15^ and *p*(ANOVA) = 8.8 × 10^−7^) (Figure 10A,B). Lastly, we monitored autophagy by analyzing the distribution of acridine orange (AO) dye using flow cytometry (Figure 10C,E). Our results demonstrated that lidocaine induced autophagy in both HepG2 and Hep3B cells, with a greater percentage of autophagic cells in HepG2 compared to Hep3B (*p*(ANOVA) = 5.4 × 10^−17^ and *p*(ANOVA) = 3.6 × 10^−13^) (Figure 10D,F). Our findings suggest that mitochondrial membrane depolarization, lipid peroxidation, and autophagy may play crucial roles as cytotoxic mechanisms in HCCs.

## 3. Discussion

Liver functional failure significantly contributes to cancer-related mortality [39,40]. While conventional chemotherapy can provide some improvement in disease control rates, progression-free survival, and overall survival, it is often accompanied by a range of adverse events. Moreover, the efficacy of cancer chemotherapy is frequently compromised by the inevitable emergence of drug resistance and various side effects [5,6,7]. This has driven ongoing research to formulate strategies that effectively tackle these challenges. One promising approach involves developing methods to minimize and prevent side effects of chemotherapy. Combination therapies often demonstrate greater effectiveness than monotherapies across various cancer types [8,9]. In our study, we investigated the use of lidocaine in combination with the well-established chemoresistant drugs cisplatin and 5-FU in HCCs. Despite the diverse characteristics of HepG2 and Hep3B cells, lidocaine displayed a synergistic effect with cisplatin and 5-FU, enhancing their cytotoxicity against HCCs. The cytotoxic effects of lidocaine may be mediated through multiple mechanisms, including the induction of ER stress, apoptosis, mitochondrial membrane depolarization, lipid peroxidation, and autophagy, while also suppressing cellular proliferation in both HepG2 and Hep3B cells. Notably, our combination index for lidocaine in conjunction with cisplatin or 5-FU was less than 1, suggesting potential clinical benefits for HCC patients undergoing HAIC or conventional chemotherapy.

Repurposing FDA-approved drugs as novel anticancer agents not only reduces the development of drug resistance but also offers significant anticancer benefits, such as decreased tumor growth, diminished metastatic potential, arrest of mitotically active cells, reduction in cancer stem cell populations, and induction of apoptosis [13,14]. Local anesthetics like lidocaine have been repurposed as anticancer agents [41,42,43]. Lidocaine has been shown to enhance the toxicity of several anticancer drugs, including mitomycin C, pirarubicin, cisplatin, and 5-FU [34,43]. The repurposing of lidocaine primarily leverages its multiple functional roles in exhibiting anticancer properties, which include suppression of cellular proliferation, invasion, and migration as well as the induction of apoptosis through various potential molecular mechanisms, such as USP14, GOLT1A, and SIRT5 [21,22,23,36]. Autophagy, which plays a “double-edged sword” role in tumorigenesis [35,44,45], can be induced by lidocaine in breast, endometrial, and bladder cancer cells [20,34,35]. In addition to apoptosis, autophagy, and the modulation of cellular proliferation, it is important to highlight lidocaine’s roles in regulating ROS, ER stress, lipid peroxidation, and mitochondrial dysfunction. The suppression of cytosolic ROS by lidocaine aligns with its observed ROS scavenging activity in dental surgery [46]. Oxidative stress arises from an imbalance between the production of ROS and the body’s antioxidant capacity [33]. Moreover, lidocaine not only suppresses ROS production but also elevates the GSH/GSSG ratio, suggesting its role as a chemosensitizer for cisplatin and 5-FU in maintaining redox homeostasis in HCCs. In contrast, lidocaine is associated with an increase in lipid peroxidation levels. These discrepancies may stem from limited access to targets for ROS and lipid peroxidation analytic agents as well as differences in subcellular localization. The balance between ROS production and antioxidant capacity remains a concern, particularly since cisplatin and 5-FU were found to increase ROS production and the GSH/GSSG ratio in our current study. Therefore, the gaps and underlying mechanisms warrant further investigation.

There are three primary reasons for pursuing combination therapies: to enhance efficacy, reduce side effects, and address cancer drug resistance [8,9]. Significantly, therapies utilizing repurposed agents have demonstrated increased effectiveness and efficiency. Cisplatin and 5-FU are recognized as the most effective chemotherapy agents for liver cancer; however, their clinical application is often limited by drug resistance, which can arise from alterations in drug transport mechanisms, increased drug inactivation, or mutations in drug targets [5,6,7]. A review of publications on PubMed regarding cisplatin resistance and 5-FU resistance revealed a total of 20,404 and 8442 publications, respectively (accessed on 5 June 2025). Platinum-based drugs like cisplatin, carboplatin, and oxaliplatin are N-guanine alkylating antitumor agents with a broad spectrum of cytotoxicity and clinical applications across various neoplastic diseases, including liver cancers. For decades, these agents have formed the cornerstone of chemotherapy and remain primary treatment options for many cancer types. 5-FU, designed to inhibit the growth of various cancers, disrupts cell progression during the S-phase and upregulates p53 [47,48]. However, chemoresistance to 5-FU presents a significant challenge for many cancers, including liver cancer. HAIC of 5-FU in combination with cisplatin has been shown to improve survival for HCC patients to an average of 14 months compared to just 5.2 months for those not receiving the combination [49]. Our current findings suggest that repurposing lidocaine may have a synergistic effect with cisplatin or 5-FU, enhancing their cytotoxicity against HCCs and indicating that lidocaine could act as a chemosensitizer in the current HAIC protocol. However, the true clinical benefit of this combination necessitates further investigation through well-structured clinical trials. In addition, one review article outlined current clinical evidence for preventive strategies against HCCs by repurposing drugs, emphasizing both established and promising avenues to effectively and safely reduce the risk of this disease [50]. The synergistic effects of lidocaine on the cytotoxicity of cisplatin or 5-FU in HCCs likely arise from mechanisms distinct from those of traditional alkylating agents or antimetabolites. Lidocaine may influence redox homeostasis, autophagy, ER stress, and mitochondrial dysfunction, which could serve as potential targets for further exploration of the underlying molecular mechanisms in HCCs. Understanding these interactions may provide insights for enhancing repurposing and combination strategies in cancer treatment. In the future, doctors can better optimize therapeutic regimens, potentially leading to improved patient outcomes in HCCs.

## 4. Materials and Methods

### 4.1. Cell Culture and Reagents

HepG2 and Hep3B HCC cell lines were obtained from the American Type Culture Collection (ATCC; Manassas, VA, USA). The cells were cultured in Dulbecco’s Modified Eagle Medium (DMEM) supplemented with 10% fetal bovine serum (FBS) and 1% Penicillin/Streptomycin solution (Invitrogen, Waltham, MA, USA), as previously described [51]. Lidocaine, cisplatin, 5-FU, thiazolyl blue tetrazolium bromide (MTT), propidium iodide (PI), 2′,7-dichlorofluorescein diacetate (DCFH-DA), and acridine orange (Cat. No. A8097) were purchased from Sigma Aldrich (St. Louis, MO, USA).

### 4.2. Cell Viability Analysis and Combination Index Calculated

HepG2 and Hep3B cells were seeded in a 96-well culture plate at a density of 1.5 × 10^4^ cells per well and treated the following day with the specified concentrations of lidocaine, cisplatin, and 5-FU for 24 h. After treatment, the cells were incubated with MTT solution for 1 h at 37 °C. Dimethyl sulfoxide (DMSO) was added to dissolve the formazan crystals, and the absorbance was measured at 570 nm and 650 nm using a multimode microplate reader (Varioskan™ LUX, Thermo Scientific™, Waltham, MA, USA). Cell viability was determined by calculating the absorbance ratio between the control group (defined as 100% cell survival) and the treatment groups. The combination index (CI) was computed using CalcuSyn software (Biosoft, Cambridge, UK) to generate the isobologram (ED50). Generally, a CI value of less than 1 indicates a synergistic combination effect, while a CI value greater than 1 suggests an additive combination effect [28].

### 4.3. Fluorescence-Activated Cell Sorting (FACS), Cell Proliferation, Cell Cycle Profiling, Apoptosis, ROS, Mitochondrial Membrane Potential Analysis, Lipid Peroxidation, and Acidic Vesicular Organelles Detection

Cell cycle profiles were obtained by measuring the cellular DNA content via flow cytometry. The cells were again seeded in 6-well culture plates and treated with the indicated concentrations of lidocaine, cisplatin, and 5-FU for 24 h. The cells were then fixed in 70% ice-cold ethanol and stored at −30 °C overnight. Afterward, they were washed twice with ice-cold PBS supplemented with 1% FBS and stained with propidium iodide (PI) solution (5 µg/mLPI in PBS, 1% Tween 20, and 0.5 µg/mL RNase A) for 30 min at 37 °C in the dark. The cell cycle distribution was subsequently evaluated using a FACSCalibur flow cytometer and CellQuest Pro software, v 6.1 (BD Biosciences, Franklin Lakes, NJ, USA), as previously described [51].

Cell proliferation was assessed using immunofluorescent staining with bromodeoxyuridine (BrdU) from the BD Pharmingen™ BrdU Flow Kit, along with flow cytometry, following the manufacturer’s instructions. In brief, HepG2 and Hep3B cells were seeded in 6-well culture plates and treated with the specified concentrations of lidocaine, cisplatin, and 5-FU for 24 h. After incubation, the cells were stained with BrdU, harvested, washed with PBS, and then fixed and permeabilized. Subsequently, the cells were labeled with fluorescent antibodies against BrdU and resuspended in a 7-AAD (7-amiono-actinomycin D) solution to stain total DNA for cell cycle analysis. The cells were analyzed for FITC-BrdU fluorescence using a FACSCalibur flow cytometer and CellQuest Pro software (BD Biosciences), as previously described [52].

Early and late stages of apoptosis were analyzed using a fluorescein Phycoerythrin (PE)-Annexin V Apoptosis Detection Kit (BD Biosciences). Following the manufacturer’s protocol, cells were stained with PE-Annexin V and 7-Amino-Actinomycin to assess the effects of lidocaine on early apoptosis (PE-Annexin-positive and 7-Amino-Actinomycin-negative), late apoptosis (PE-Annexin-positive and 7-Amino-Actinomycin-positive), and necrosis (PE-Annexin-negative and 7-Amino-Actinomycin-positive). The analysis was performed using a FACSCalibur flow cytometer and CellQuest Pro software (BD Biosciences), as previously described [52].

Intracellular ROS levels were measured using the fluorescent marker DCFH-DA. Cells were treated with varying concentrations of lidocaine, in combination with cisplatin and 5-FU for 24 h, and stained with DCFH-DA (10 μM) for 40 min at 37 °C. After washing the cells with PBS, fluorescence was analyzed on channel FL-1 of the FACSCalibur flow cytometer using CellQuest Pro software (BD Biosciences). The gating strategy for cell volume involved forward scatter height (FSC-H) and side scatter height (SSC-H), with the median fluorescence intensity of the vehicle serving as the basis for M2 gating, as previously described [53].

Mitochondrial depolarization was quantified by measuring the red/green fluorescence intensity ratio. All cells, both dead and viable, were harvested, washed with PBS, and incubated with 1× binding buffer containing the mitochondrial membrane potential-sensitive fluorescent dye JC-1 (BD^TM^ MitoScreen, BD Biosciences) for 30 min at 37 °C in the dark. Following a wash with PBS, JC-1 fluorescence was analyzed on channels FL-1 and FL-2 of the FACSCalibur flow cytometer using CellQuest Pro software (BD Biosciences) to detect the monomer (green fluorescence) and aggregate (red fluorescence) forms of the dye, as previously described [51].

The status of cellular lipid peroxidation was assessed using the BODIPY™ 581/591 C11 (Invitrogen™) fluorescent dye. Cells were exposed to various concentrations of lidocaine for 24 h, harvested, and stained with BODIPY-C11 581/591 (10 µM) for 30 min at 37 °C. After washing the cells with PBS, fluorescence intensity was analyzed on channel FL-1 of the FACSCalibur flow cytometer using CellQuest Pro software (BD Biosciences). The BODIPY-C11 dye exhibits a peak excitation and emission of 581/591 nm in its reduced state, while oxidation shifts the fluorescence to 488/510 nm, as previously described [54].

Lastly, acidic cellular compartments were detected using acridine orange staining and analyzed by flow cytometry. As the protonated form of acridine orange accumulates in acidic vesicles, it serves as a marker for the later stages of autophagy. Cells were treated with the indicated doses of lidocaine for 24 h, stained with acridine orange (1 μg/)mL for 20 min at 37 °C, and then trypsinized for harvesting. The cells were washed with PBS, resuspended in 400 µL of PBS, and analyzed via flow cytometry (FACSCalibur, BD Biosciences). The excitation wavelength was set at 488 nm, and fluorescence was detected at 510–530 nm (green fluorescence, FL1) and 650 nm (red fluorescence, FL3). Data analysis was conducted using the CellQuest™ software program, and the percentage of autophagic cells was calculated based on the number of cells in the upper-left and upper-right quadrants, as previously described [53].

### 4.4. GSH/GSSG Measurement

Changes in the GSH/GSSG ratio have been associated with various human diseases, aging, and cell signaling events. To assess these alterations, the GSH/GSSG-Glo™ Assay (Promega, Madison, WI, USA) was employed, following the manufacturer’s instructions. Briefly, HepG2 and Hep3B cells were seeded in a 96-well culture plate at a density of 1.5 × 10^4^ cells per well and treated the following day with lidocaine, cisplatin, and 5-FU for 24 h. After treatment, the cells were washed with PBS and subsequently exposed to 50 µL of either total GSH lysis reagent (for total GSH measurements) or oxidized GSH lysis reagent (for GSSG measurements). Following this, 50 µL of Luciferin Generation Reagent was added to each well, mixed, and incubated for 30 min. Then, 100 µL of Luciferin Detection Reagent was added to all wells, mixed thoroughly, and incubated for an additional 15 min before measuring luminescence. The GSH/GSSG ratio was calculated from the luminescence values, expressed in relative light units (RLU), using the formula (total GSH RLU—GSSG RLU)/(GSSG RLU/2), as previously described [55].

### 4.5. Western Blotting Analysis

Cells were lysed with lysis buffer (100 mM Tris-HCl (pH 8.0), 150 mM NaCl, 0.1% SDS, and 1% Triton 100) at 4 °C. Protein concentrations in the lysates were measured using Bio-Rad Protein Assay Dye Reagent Concentrate (Bio-Rad Laboratories, Hercules, CA, USA). Protein lysate (30 μg) were prepared with 4× protein loading dye and denatured at 95 °C for 10 min, separated on 12% SDS-PAGE, and blotted onto PVDF membranes (Immobilon-P; Millipore, Bedford, MA, USA) using a Bio-Rad Semi-Dry Transfer Cell, and the membranes were blocked with 5% nonfat milk in shaking. The blots were then incubated with primary antibodies against α-actinin (ACTN) (H-2) and p62 (D-3) (Santa Cruz Biotechnology, Santa Cruz, CA, USA); CHOP (2895), LC3B (2775), H3 (9715), and EGFR (4267) (Cell Signaling Technology, Danvers, MA, USA); and Cyclin D1 (ab134175) and p21 (ab109520) (Abcam, Cambridge, UK). Thereafter, the blots were incubated with KPL peroxidase-labeled antibodies (anti-mouse IgG, 5220-0341, 074-1806; and anti-rabbit IgG, 5220-0336, 074-1506, Seracare, Milford, MA, USA). The immunoreactive proteins were detected using ECL^TM^ Western Blotting Detection Reagent and Amersham Hyperfilm^TM^ ECL (GE Healthcare, Chicago, IL, USA), as previously described [52].

### 4.6. Statistical Analysis

The values are expressed as the means ± SDs of at least three independent experiments. All the comparisons between groups were conducted using Student’s *t*-tests, and the comparison between multiple groups was conducted using analysis of variance (ANOVA) with SPSS 20.0 for Windows (SPSS, Chicago, IL, USA). The statistical significance was set to *p <* 0.05.

## 5. Conclusions

In our study, we investigated the cytotoxic effects of lidocaine when combined with cisplatin or 5-FU on HepG2 and Hep3B cell lines. Our findings indicate that lidocaine can synergistically enhance the cytotoxic effects of cisplatin and 5-FU in HCCs. The mechanisms underlying these cytotoxic effects may involve the induction of ER stress, initiation of apoptosis, mitochondrial membrane depolarization, lipid peroxidation, autophagy, and the suppression of cellular proliferation in both HepG2 and Hep3B cells. Thus, our study highlights the promising synergistic potential of combining lidocaine with cisplatin or 5-FU for the treatment of HCCs, suggesting that lidocaine may serve as an effective chemosensitizer in this context.

## Data Availability

The raw data supporting the conclusions of this article will be made available by the authors on request.

## Supplementary Materials

The following supporting information can be downloaded at: https://www.mdpi.com/article/10.3390/ijms26157137/s1.
