# Supplementary material for: Chemosensitizer Effects of Cisplatin- and 5-Fluorouracil-Treated Hepatocellular Carcinomas by Lidocaine
Source: Int J Mol Sci. 2025 Jul 24;26(15):7137. doi: 10.3390/ijms26157137 (PMC12345720; doi:10.3390/ijms26157137)
Supplement: Supplementary file 1 [file ijms-26-07137-s001.zip › ijms-3724681-supplementary.pdf]

	Lidocaine					
	0 mM	1 mM	2 mM	3 mM	4 mM	5 mM
	1.4376	1.2829	1.0469	0.8076	0.5031	0.0729
	1.4489	1.2855	1.0556	0.8314	0.5474	0.0762
	1.46	1.2634	1.0484	0.8679	0.51	0.0763
Average	1.4488333	1.2772667	1.0503	0.8356333	0.5201667	0.0751333
Avedev	0.0074889	0.0092444	0.0035333	0.0215111	0.0181556	0.0014889
Ratio	100	88.15829	72.492812	57.676293	35.902451	5.1857818
	0.516891	0.6380613	0.2438744	1.4847195	1.2531156	0.1027647

	Lidocaine					
dose	0	1	2	3	4	5
Average	100	88.15829	72.492812	57.676293	35.902451	5.1857818
Avedev	0.516891	0.6380613	0.2438744	1.4847195	1.2531156	0.1027647

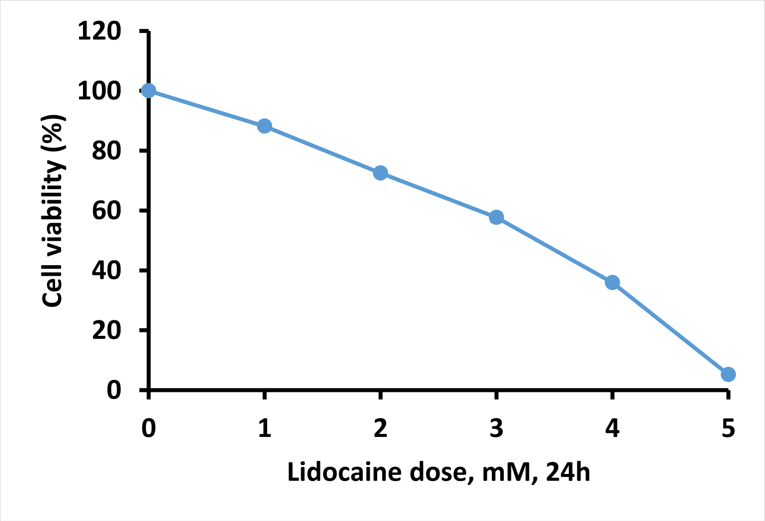

Figure S1A. Effects of lidocaine on cell viability in HepG2 cells. HepG2 cells were treated with the specified concentrations of lidocaine for 24 hours.

	Cisplatin					
	0 uM	5 uM	10 uM	20 uM	50 uM	80 uM
	1.4479	1.3537	1.2098	0.7398	0.7366	0.6525
	1.4326	1.3311	1.2007	0.7867	0.6906	0.6185
	1.3902	1.3719	1.1822	0.7955	0.6722	0.58
Average	1.4235667	1.3522333	1.1975667	0.774	0.6998	0.617
Avedev	0.0222444	0.0140889	0.0102444	0.0228	0.0245333	0.0246667
Ratio	100.00000	94.98911	84.12438	54.37048	49.15822	43.34184
	1.56259	0.98969	0.71963	1.60161	1.72337	1.73274

	Cisplatin					
dose	0	5	10	20	50	80
Average	100.0	95.0	84.1	54.4	49.2	43.3
Avedev	1.56259	0.98969	0.71963	1.60161	1.72337	1.73274

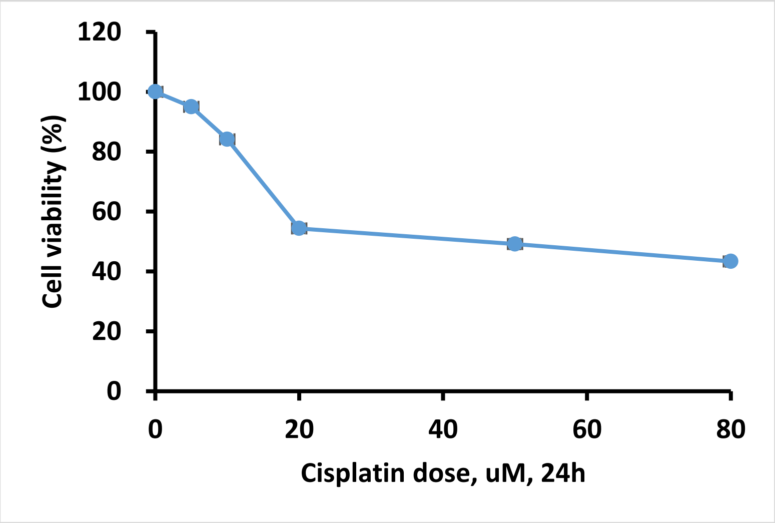

Figure S1B. Effects of cisplatin on cell viability in HepG2 cells. HepG2 cells were treated with the specified concentrations of cisplatin for 24 hours.

	5-FU					
	0 uM	10 uM	20 uM	50 uM	100 uM	200 uM
	1.3653	1.1278	1.3215	1.3293	1.2923	1.1379
	1.4473	1.1125	1.3288	1.2978	1.3288	1.102
	1.3875	1.0669	1.3225	1.3414	1.3691	1.113
Average	1.4000333	1.1024	1.3242667	1.3228333	1.3300667	1.1176333
Avedev	0.0315111	0.0236667	0.0030222	0.0166889	0.0260222	0.0135111
Ratio	100	78.740984	94.588226	94.485848	95.002502	79.829054
	2.2507401	1.690436	0.2158679	1.1920351	1.8586859	0.9650564

	5-FU					
dose	0	10	20	50	100	200
Average	100	78.740984	94.588226	94.485848	95.002502	79.829054
Avedev	2.2507401	1.690436	0.2158679	1.1920351	1.8586859	0.9650564

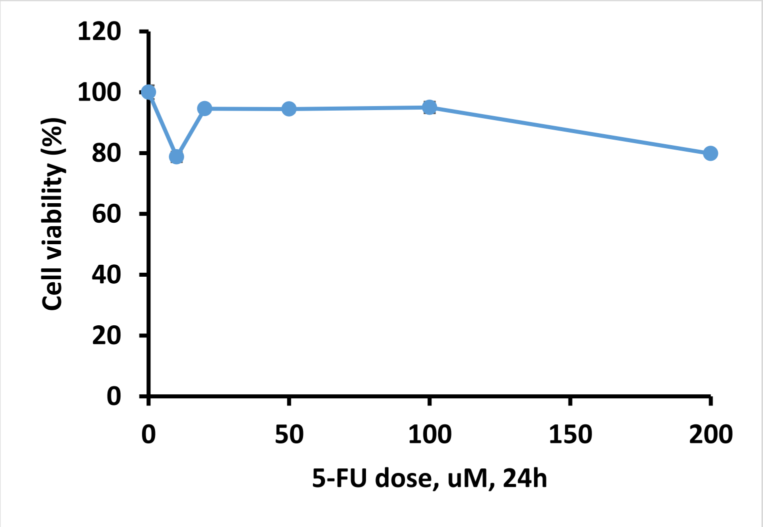

Figure S1C. Effects of 5-FU on cell viability in HepG2 cells. HepG2 cells were treated with the specified concentrations of 5-FU for 24 hours.

	Lidocaine					
	0 mM	1 mM	2 mM	3 mM	4 mM	5 mM
	2.1556	1.9458	2.0276	1.7924	1.5339	1.1329
	2.0169	1.758	1.7449	1.7749	1.5319	1.0425
	2.0576	1.9071	1.7001	1.6796	1.5893	1.1411
Average	2.0767	1.8703	1.8242	1.7489667	1.5517	1.1055
Avedev	0.0526	0.0748667	0.1356	0.0462444	0.0250667	0.042
Ratio	100	90.061155	87.841287	84.218552	74.719507	53.233495
	2.5328646	3.6050786	6.5295902	2.2268235	1.2070432	2.0224394

	Lidocaine					
dose	0	1	2	3	4	5
Average	100	90.061155	87.841287	84.218552	74.719507	53.233495
Avedev	2.5328646	3.6050786	6.5295902	2.2268235	1.2070432	2.0224394

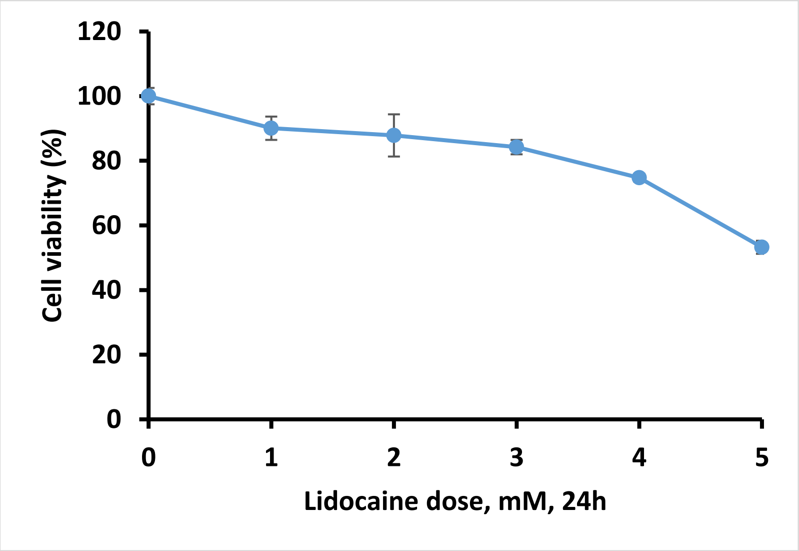

Figure S2A. Effects of lidocaine on cell viability in Hep3B cells. Hep3B cells were treated with the specified concentrations of lidocaine for 24 hours.

	Cisplatin					
	0 uM	5 uM	10 uM	20 uM	50 uM	80 uM
	1.9222	1.7888	1.7393	1.3251	1.0001	0.5127
	1.9884	1.8728	1.7417	1.3522	0.9325	0.5304
	2.1256	1.9025	1.7414	1.3475	0.943	0.5032
Average	2.0120667	1.8547	1.7408	1.3416	0.9585333	0.5154333
Avedev	0.0756889	0.0439333	0.001	0.011	0.0277111	0.0099778
Ratio	100.0000	92.1789	86.5180	66.6777	47.6392	25.6171
	3.7617	2.1835	0.0497	0.5467	1.3772	0.4959

	Cisplatin					
dose	0	5	10	20	50	80
Average	100.0000	92.1789	86.5180	66.6777	47.6392	25.6171
Avedev	3.7617	2.1835	0.0497	0.5467	1.3772	0.4959

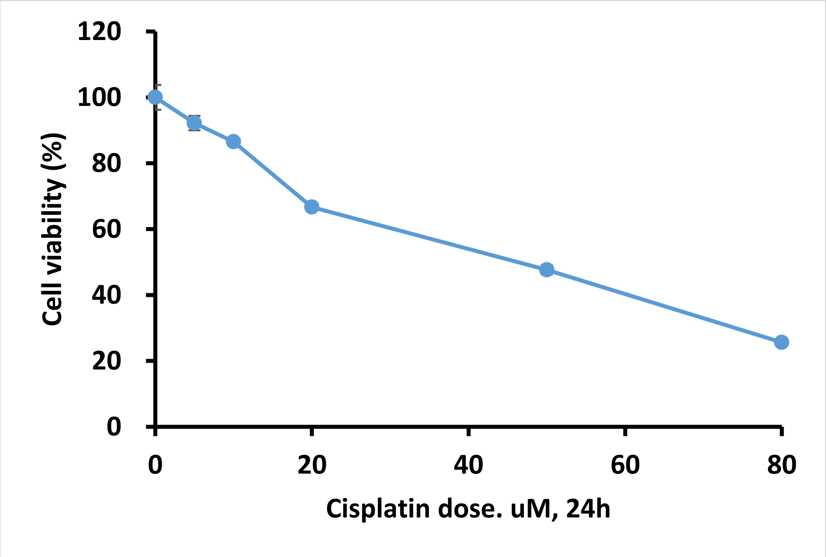

Figure S2B. Effects of cisplatin on cell viability in Hep3B cells. Hep3B cells were treated with the specified concentrations ofcisplatin for 24 hours.

5-FU

	0 uM	10 uM	20 uM	50 uM	100 uM	200 uM
	1.9339	1.7707	1.7047	1.7802	1.7222	1.5475
	1.9189	1.8946	1.7652	1.7459	1.7645	1.6735
	1.986	1.7957	1.7922	1.7799	1.7943	1.64
Average	1.9462667	1.8203333	1.7540333	1.7686667	1.7603333	1.6203333
Avedev	0.0264889	0.0495111	0.0328889	0.0151778	0.0254222	0.0485556
Ratio	100.0000	93.5295	90.1230	90.8748	90.4467	83.2534
	1.3610	2.5439	1.6898	0.7798	1.3062	2.4948

	5-FU					
dose	0	10	20	50	100	200
Average	100.000	93.529	90.123	90.875	90.447	83.253
Avedev	1.361	2.544	1.690	0.780	1.306	2.495

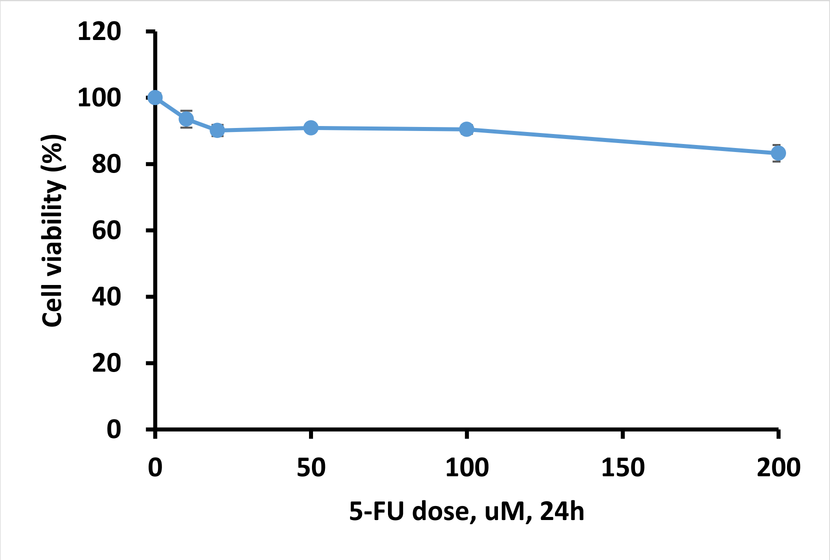

Figure S2C. Effects of 5-FU on cell viability in Hep3B cells. Hep3B cells were treated with the specified concentrations of 5-FU for 24 hours.

570 nm- 650 nm		Cisplatin dose, 24h 0 uM	0.156 uM	0.3125 uM	0.625 uM	1.25 uM	2.5 uM	5 uM	10 uM	20 uM	40 uM
Lidocaine c	0 mM	1.1946	1.1607	1.1119	1.1865	1.1082	1.0455	1.0615	0.8613	0.6701	0.6538
	1 mM	1.0282	1.0391	1.0154	1.0055	1.005	0.9644	1.0041	0.8557	0.7105	0.516
	2 mM	0.9063	0.915	0.9073	0.8387	0.8506	0.8215	0.8577	0.7385	0.6078	0.1811
	3 mM	0.6686	0.6655	0.68	0.7128	0.6094	0.6731	0.6616	0.5455	0.3021	0.1853
	4 mM	0.5705	0.4978	0.4983	0.4787	0.5	0.4868	0.4742	0.3842	0.167	0.4117
	5 mM	0.5337	0.5612	0.4885	0.5341	0.4606	0.4574	0.4873	0.3994	0.393	0.2288
	6 mM	0.0825	0.109	0.115	0.1109	0.0985	0.1155	0.08	0.0922	0.0419	0.0288
	7 mM	0.0157	0.0166	0.0173	0.0177	0.0174	0.0168	0.0155	0.0152	0.0152	0.013

Drug: lido

Data points entered:

lido	Fraction affected
1	0.1409
2	0.1004
2	0.244
3	0.1407
3	0.4452
4	0.2641
4	0.5282
5	0.6037
5	0.5594
6	0.8339
6	0.9412
7	0.9985
7	0.9977

Median-effect parameters:

	lido
Dm:	3.05051
lower 95%:	2.27E+00
upper 95%	4.09306
m:	3.73695 +/- 0.949416
y-int:	-1.8101 +/- 0.587507
r:	0.76471
CCSI:	-0.92049

Drug: cisplatin

Data points entered:

cisplatin	Fraction affected
0.156	0.0288
0.3125	7.01E-02
0.625	0.0069
1.25	0.0732
2.5	0.1263
5	0.1127
10	0.2821
20	0.4439
40	0.4577

Median-effect parameters:

	cisplatin
Dm:	51.00931
lower 95%:	10.41621
upper 95%	19:09
m:	0.69761 +/- 0.161227
y-int:	-1.1913 +/- 0.140795
r:	0.85315
CCSI:	-0.4556

Drug: MAX:MAX

Combinatic lido cisplatin

Ratio: 40:00.0

Data points entered:

lido ()	cisplatin ()	Fraction affected
1	5.71429	0.1601
2	11.4286	0.2912
3	17.1429	0.4414
4	22.8571	0.6097
5	28.5714	0.673
6	34.2857	0.9755
7	40	1

Median-effect parameters:

	lido ()	cisplatin ()
Dm:	2.24369	12.82108
lower 95%:	1.17E+00	6.68512
upper 95%	4.30307	24.58897
m:	4.79408 +/- 2.109970	
y-int:	-1.6825 +/- 1.258173	
r:	0.71274	
CCSI:	-0.887	

CI Simulations

Fa	CI	Est. s.d.	lido ()	cisplatin ()
0.02	30.475	30.4211	0.99633	5.69332
0.05	1.01E+01	7.41E+00	1.21402	6.93727
0.1	4.55E+00	2.74E+00	1.41878	8.10734
0.15	2.919	1.63E+00	1.56252	8.92869
0.2	2.171	1.162	1.68027	9.60153
0.25	1.75	0.9039	1.78418	10.19534
0.3	1.483	0.7394	1.88021	10.74405
0.35	1.299	0.6243	1.9719	11.26798
0.4	1.166	0.5386	2.06173	11.78131
0.45	1.066	0.472	2.15171	12.29549
0.5	0.987	0.4186	2.24369	12.82108
0.55	0.923	0.3748	2.3396	13.36914
0.6	0.871	0.3383	2.44171	13.95262
0.65	0.827	0.3076	2.55294	14.58826
0.7	0.789	0.2817	2.67744	15.29964
0.75	0.755	0.26	2.82154	16.12307
0.8	0.724	0.2424	2.99604	17.1202
0.85	0.694	0.2291	3.22181	18.41034
0.9	0.663	0.2219	3.54821	20.27547
0.95	0.625	0.2267	4.14667	23.69523
0.99	5.62E-01	0.2717	5.85105	33.43457

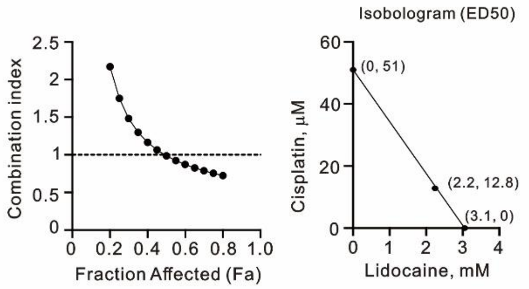

Figure S3A Combination index of lidocaine with cisplatin in HepG2 cells. The combination index for lidocaine in conjunction with cisplatin was calculated. Additionally, Isobolograms (ED_{50}) for the combinations of lidocaine with cisplatin or 5-FU were determined using CalcuSyn software.

570 nm- 650 nm	5-FU dose, 24h<br>0 uM	5 uM	10 uM	20 uM	50 uM	100 uM	200 uM	250 uM	350 uM	500 uM
Lidocaine c 0 mM	1.1281	1.0427	1.0702	1.0828	1.0606	0.9362	1.0532	0.9926	0.8941	0.7622
1 mM	1.1341	1.0386	1.0471	1.0396	1.0036	0.995	1.0621	1.0731	1.0394	0.9526
2 mM	1.0215	1.0328	1.0208	1.0876	0.9456	0.9884	0.9294	1.0363	1.0044	0.9631
3 mM	0.9762	0.9426	0.9864	0.9682	0.9562	0.8667	0.8045	0.9387	0.9623	0.8536
4 mM	0.8377	0.7933	0.7767	0.8463	0.7601	0.8416	0.7275	0.77	0.7138	0.5962
5 mM	0.4565	0.5153	0.5112	0.553	0.4389	0.4819	0.4512	0.441	0.4119	0.3218
6 mM	0.198	0.2139	0.1898	0.2034	0.1796	0.1954	0.1599	0.1508	0.1387	0.127
7 mM	0.0133	0.0135	0.015	0.0158	0.0138	0.015	0.0132	0.0131	0.0118	0.0116

Drug: lido

Data points entered:

lido	Fraction affected
1	0.1409
2	0.1004
2	0.244
3	0.1407
3	0.4452
4	0.2641
4	0.5282
5	0.6037
5	0.5594
6	0.8339
6	0.9412
7	0.9985
7	0.9977

Median-effect parameters:

	lido
Dm:	3.05051
lower 95%:	2.27E+00
upper 95%	4.09306
m:	3.73695 +/- 0.949416
y-int:	-1.8101 +/- 0.587507
r:	0.76471
CCSI:	-0.92049

Drug: 5-FU

Data points entered:

5-FU	Fraction affected
200	0.0721
250	1.26E-01
350	0.2139
500	0.3314

Median-effect parameters:

	5-FU
Dm:	692.8752
lower 95%:	608.1357
upper 95%	789.4226
m:	1.98516 +/- 0.148752
y-int:	-5.6392 +/- 0.370400
r:	0.99443
CCSI:	########

Drug: MAX:MAX

Combinatic lido 5-FU

Ratio: 01:20

Data points entered:

lido<br>()	5-FU<br>()	Fraction affected
1	00:00.0	0.0843
2	40	0.168
3	60	0.2383
4	80	0.3623
5	100	0.6175
6	120	0.8868
7	140	1

Median-effect parameters:

	lido<br>()	5-FU<br>()
Dm:	2.63394	52.67876
lower 95%:	1.40253	28.05059
upper 95%	4.94651	98.93024
m:	4.84146 +/- 2.284853	
y-int:	-2.0363 +/- 1.362456	
r:	6.88E-01	
CCSI:	-0.887	

CI Simulations

Fa	CI	Est. s.d.	lido<br>()	5-FU<br>()
0.02	1.337	9.06E-01	1.18E+00	23.57909
0.05	1.216	0.7056	1.43E+00	28.67545
0.1	1.133	0.5775	1.67305	33.4609
0.15	1.087	5.10E-01	1.8408	36.81598
0.2	1.054	0.4648	1.97811	39.56222
0.25	1.029	0.4312	2.09921	41.98427
0.3	1.01E+00	4.05E-01	2.21106	44.22117
0.35	9.88E-01	3.83E-01	2.3178	46.35598
0.4	0.971	3.64E-01	2.42233	48.44668
0.45	0.955	0.3478	2.527	50.53994
0.5	0.939	0.3337	2.63394	52.67876
0.55	0.925	0.3213	2.7454	54.90809
0.6	0.91	0.3105	2.86403	57.28053
0.65	0.895	0.301	2.9932	59.86394
0.7	0.879	0.2931	3.1377	62.75391
0.75	0.862	0.2868	3.30487	66.09742
0.8	0.844	0.2826	3.5072	70.14399
0.85	0.822	0.2815	3.76881	75.37627
0.9	0.795	0.2859	4.14671	82.93415
0.95	0.753	0.3037	4.83872	96.77449
0.99	0.672	0.367	6.80459	136.0918

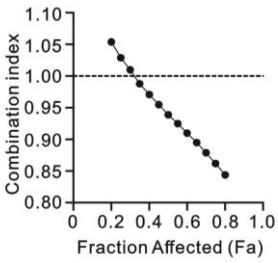

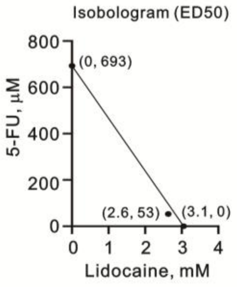

Figure S3B Combination index of lidocaine with 5-FU in HepG2 cells. The combination index for lidocaine in conjunction with 5-FU was calculated. Additionally, Isobolograms (ED_{50}) for the combinations of lidocaine with cisplatin or 5-FU were determined using CalcuSyn software.

570 nm- 650 nm	Cisplatin dose, 24h<br>0 uM	0.156 uM	0.3125 uM	0.625 uM	1.25 uM	2.5 uM	5 uM	10 uM	20 uM	40 uM
Lidocaine c 0 mM	1.1986	1.16	1.2004	1.2054	1.1836	1.1041	1.0705	0.9538	0.7231	0.4789
1 mM	1.1614	1.1231	1.1821	1.113	1.0871	1.0505	1.0199	1.0002	0.6525	0.3882
2 mM	1.0638	1.0704	1.0728	1.1005	1.0302	0.9984	0.938	0.7539	0.5157	0.2239
3 mM	0.9654	1.0111	1.0124	1.1233	0.9988	0.9063	0.7877	0.6112	0.3685	0.1295
4 mM	0.9151	0.9447	0.9225	0.9742	0.8692	0.8088	0.5967	0.3933	0.2224	0.0758
5 mM	0.6156	0.6379	0.5894	0.6051	0.5354	0.4945	0.2881	0.1839	0.1359	0.0454
6 mM	0.2252	0.2621	0.2565	0.229	0.2044	0.1717	0.1185	0.0918	0.0643	0.0295
7 mM	0.0179	0.0205	0.0218	0.0192	0.0199	0.016782	0.0178	0.0142	0.0138	0.0118

Drug: Lido

Data points entered:

Lido	Fraction affected
4	0.2641
4	0.2433
5	0.6037
5	0.4942
6	0.8339
6	0.8212
7	0.9985
7	0.9949

Median-effect parameters:

	Lido
Dm:	4.67204
lower 95%:	4.27693
upper 95%	5.10364
m:	11.57942 +/- 2.068408
y-int:	-7.7525 +/- 1.523740
r:	9.16E-01
CCSI:	-0.99239

Drug: cisplatin

Data points entered:

cisplatin	Fraction affected
0.156	0.0381
0.3125	0.0043
0.625	7.54E-05
1.25	0.0183
2.5	0.0849
5	0.1131
10	2.11E-01
20	0.4041
40	0.6087

Median-effect parameters:

	cisplatin
Dm:	36.26585
lower 95%:	3.96881
upper 95%	331.3867
m:	1.15723 +/- 0.405865
y-int:	-1.8047 +/- 0.354432
r:	0.73303
CCSI:	########

Drug: MAX:MAX

Combinatic Lido cisplatin

Ratio: 01:00.6

Data points entered:

	Lido<br>()	cisplatin<br>()	Fraction affected
	1	00:00.0	0.0775
	2	1.25	0.1468
	3	1.875	0.2506
	4	2.5	0.51
	5	3.125	0.8558
	6	3.75	0.956
	7	4.375	1

Median-effect parameters:

	Lido<br>()	cisplatin<br>()
Dm:	2.50235	1.56397
lower 95%:	1.46433	0.9152
upper 95%	4.27621	2.67263
m:	5.34938 +/- 2.084173	
y-int:	-2.1309 +/- 1.242791	
r:	7.54E-01	
CCSI:	-0.887	

CI Simulations

Fa	CI	Est. s.d.	Lido<br>()	cisplatin<br>()
0.02	0.964	6.42E-01	1.21E+00	0.75556
0.05	0.715	0.3726	1.44E+00	0.90195
0.1	0.62	0.2763	1.65945	1.03716
0.15	0.589	2.40E-01	1.80935	1.13084
0.2	0.576	0.2194	1.93108	1.20693
0.25	0.57	0.2058	2.03778	1.27361
0.3	5.68E-01	1.96E-01	2.13579	1.33487
0.35	5.69E-01	1.88E-01	2.2289	1.39306
0.4	0.571	1.82E-01	2.31969	1.44981
0.45	0.574	0.1768	2.41022	1.50639
0.5	0.579	0.1727	2.50235	1.56397
0.55	0.584	0.1692	2.598	1.62375
0.6	0.591	0.1665	2.69939	1.68712
0.65	0.598	0.1644	2.80935	1.75584
0.7	0.608	0.1631	2.93182	1.83239
0.75	0.619	0.1628	3.07284	1.92053
0.8	0.633	0.1638	3.24262	2.02664
0.85	0.651	0.1673	3.46078	2.16299
0.9	0.678	0.1756	3.77339	2.35837
0.95	0.726	0.1984	4.33906	2.71191
0.99	0.852	0.2902	5.90755	3.69222

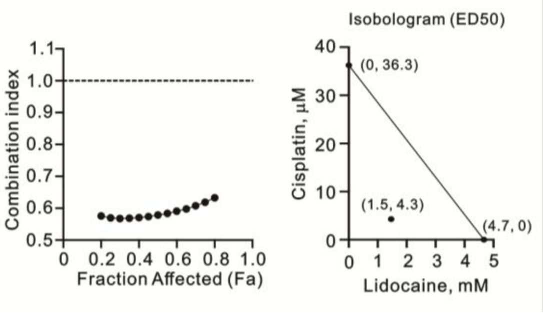

Figure S3C Combination index of lidocaine with cisplatin in Hep3B cells. The combination index for lidocaine in conjunction with cisplatin was calculated. Additionally, Isobolograms (ED_{50}) for the combinations of lidocaine with cisplatin or 5-FU were determined using CalcuSyn software.

570 nm- 650 nm		5-FU dose, 24h 0 uM	5 uM	10 uM	20 uM	50 uM	100 uM	200 uM	250 uM	350 uM	500 uM
Lidocaine (	0 mM	1.1281	1.0427	1.0702	1.0828	1.0606	0.9362	1.0532	0.9926	0.8941	0.7622
	1 mM	1.1341	1.0386	1.0471	1.0396	1.0036	0.995	1.0621	1.0731	1.0394	0.9526
	2 mM	1.0215	1.0328	1.0208	1.0876	0.9456	0.9884	0.9294	1.0363	1.0044	0.9631
	3 mM	0.9762	0.9426	0.9864	0.9682	0.9562	0.8667	0.8045	0.9387	0.9623	0.8536
	4 mM	0.8377	0.7933	0.7767	0.8463	0.7601	0.8416	0.7275	0.77	0.7138	0.5962
	5 mM	0.4565	0.5153	0.5112	0.553	0.4389	0.4819	0.4512	0.441	0.4119	0.3218
	6 mM	0.198	0.2139	0.1898	0.2034	0.1796	0.1954	0.1599	0.1508	0.1387	0.127
	7 mM	0.0133	0.0135	0.015	0.0158	0.0138	0.015	0.0132	0.0131	0.0118	0.0116

Title:
Date:
Investigator:
Filename: CalcuSyn2

Drug: Lido

Data points entered:

Lido	Fraction affected
4	0.2641
4	0.2433
5	0.6037
5	0.4942
6	0.8339
6	0.8212
7	0.9985
7	0.9949

Median-effect parameters:
Lido

Dm: 4.67204
lower 95%: 4.27693
upper 95% 5.10364

m: 11.57942 +/- 2.068408
y-int: -7.7525 +/- 1.523740
r: 9.16E-01
CCSI: -0.99239

Drug: 5-FU

Data points entered:

5-FU	Fraction affected
10	0.057
20	0.0458
50	0.0656
100	0.1764
350	0.2139
500	0.3314

Median-effect parameters:
5-FU

Dm: 2435.429
lower 95%: 624.6309
upper 95% 9495.712

m: 0.57634 +/- 0.106418
y-int: -1.9518 +/- 0.209870
r: 0.93808
CCSI: -0.95016

0
Drug: MAX:MAX
Combinatio Lido 5-FU
Ratio: 01:20

Data points entered:

	Lido ()	5-FU ()	Fraction affected
	1	20	0.0843
	2	960:00:00	0.168
	3	60	0.2383
	4	00:00.0	0.3623
	5	100	0.6175
	6	120	0.8868
	7	140	1

Median-effect parameters:

	Lido ()	5-FU ()
Dm:	2.63394	52.67876
lower 95%:	1.40253	28.05059
upper 95%:	4.94651	98.93024

m: 4.84146 +/- 2.284853
y-int: -2.0363 +/- 1.362456
r: 0.68784
CCSI: -0.887

CI Simulations

Fa	CI	Est. s.d.	Lido ()	5-FU ()
0.02	8.644	7.8987	1.18E+00	23.57909
0.05	2.344	1.4658	1.43E+00	28.67545
0.1	1.055	5.17E-01	1.67E+00	33.4609
0.15	0.764	3.38E-01	1.84E+00	36.81598
0.2	0.657	0.2724	1.98E+00	39.56222
0.25	0.61	0.2399	2.09921	41.98427
0.3	0.588	2.21E-01	2.21106	44.22117
0.35	0.579	0.2096	2.3178	46.35598
0.4	0.577	0.202	2.42233	48.44668
0.45	5.80E-01	1.97E-01	2.527	50.53994
0.5	5.85E-01	1.94E-01	2.63394	52.67876
0.55	0.593	1.92E-01	2.7454	54.90809
0.6	0.604	0.1916	2.86403	57.28053
0.65	0.616	0.1923	2.9932	59.86394
0.7	0.63	0.1944	3.1377	62.75391
0.75	0.647	0.1983	3.30487	66.09742
0.8	0.669	0.205	3.5072	70.14399
0.85	0.696	0.2161	3.76881	75.37627
0.9	0.735	0.2365	4.14671	82.93415
0.95	0.803	0.2837	4.83872	96.77449
0.99	0.979	0.4547	6.80459	136.0918

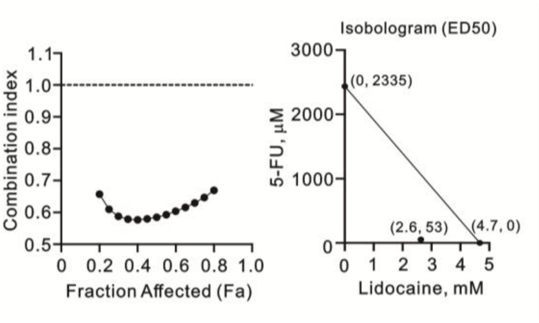

Figure S3D Combination index of lidocaine with 5-FU in Hep3B cells. The combination index for lidocaine in conjunction with 5-FU was calculated. Additionally, Isobolograms (ED_{50}) for the combinations of lidocaine with cisplatin or 5-FU were determined using CalcuSyn software.
